# Neuronal hyperexcitability in Alzheimer’s disease: what are the drivers behind this aberrant phenotype?

**DOI:** 10.1038/s41398-022-02024-7

**Published:** 2022-06-22

**Authors:** Helena Targa Dias Anastacio, Natalie Matosin, Lezanne Ooi

**Affiliations:** 1 Illawarra Health and Medical Research Institute, Wollongong, NSW 2522 Australia; 2 Molecular Horizons and School of Chemistry and Molecular Bioscience, University of Wollongong, Wollongong, NSW 2522 Australia

**Keywords:** Molecular neuroscience, Diseases

## Abstract

Alzheimer’s disease (AD) is a progressive neurodegenerative disorder leading to loss of cognitive abilities and ultimately, death. With no cure available, limited treatments mostly focus on symptom management. Identifying early changes in the disease course may provide new therapeutic targets to halt or reverse disease progression. Clinical studies have shown that cortical and hippocampal hyperactivity are a feature shared by patients in the early stages of disease, progressing to hypoactivity during later stages of neurodegeneration. The exact mechanisms causing neuronal excitability changes are not fully characterized; however, animal and cell models have provided insights into some of the factors involved in this phenotype. In this review, we summarize the evidence for neuronal excitability changes over the course of AD onset and progression and the molecular mechanisms underpinning these differences. Specifically, we discuss contributors to aberrant neuronal excitability, including abnormal levels of intracellular Ca^2+^ and glutamate, pathological amyloid β (Aβ) and tau, genetic risk factors, including *APOE*, and impaired inhibitory interneuron and glial function. In light of recent research indicating hyperexcitability could be a predictive marker of cognitive dysfunction, we further argue that the hyperexcitability phenotype could be leveraged to improve the diagnosis and treatment of AD, and present potential targets for future AD treatment development.

## Alzheimer’s disease and neuronal hyperexcitability

It is estimated that approximately 50 million people worldwide suffer from dementia [1]. Alzheimer’s disease (AD) is the most common cause of dementia, accounting for 60–80% of all cases [2]. AD causes a progressive decline in cognitive function and eventually leads to death. Amyloid plaque deposition and neurofibrillary tangles (NFT) in the brain are the main pathological hallmarks of this disease. Amyloid plaques are comprised of insoluble Aβ peptides that accumulate in the extracellular space [3], while NFTs are intraneuronal aggregates containing hyperphosphorylated and misfolded tau [4]. AD can be caused by mutations in genes involved in the processing of Aβ, including *APP*, *PSEN1*, and *PSEN2* [5]. However, familial inherited genetic cases of the disease (familial AD, FAD) represent only around 2% of total cases [6]. The majority of cases are sporadic AD or late-onset Alzheimer’s disease (LOAD), where the disease is driven by a combination of genetic and other risk factors. One of the major genetic risk factors for LOAD is the *APOE4* allele. There are three different alleles for the *APOE* gene: ε2, ε3, and ε4. The allele ε3 is the most common in the general population, and while ε4 increases the risk for AD, ε2 is protective against the disease [7]. Despite decades of research into the genetic basis and pathological mechanisms of AD, there is no treatment available to halt or reverse its progression, leading to an urgency for the development of new treatment options. Neuronal hyperexcitability can be detected, in a non-invasive manner, before the onset of dementia [8, 9]. Therefore, potentially, this phenotype could be harnessed to identify individuals at risk and start treatments before degeneration progresses too far.

Hyperexcitability can be defined as the increased likelihood that a neuron will be activated by a certain stimulus. In patients, neuronal hyperexcitability has been observed via enhanced brain activity when individuals perform a memory-encoding task [8–10]. In preclinical animal and cell models of AD, neurons show a higher frequency of action potential firing, as well as a lower threshold for firing [11–15]. Importantly, activity recordings at the level of single neurons, neuronal networks or even entire brain regions consistently show hyperexcitability in the early stages of AD (summarized in Table 1). This evidence comes from laboratory models of AD, as well as living patients, showcasing the potential of neuronal excitability changes as a biomarker for early detection of AD. Beyond that, the molecular mechanisms driving neuronal hyperexcitability are also potential targets for therapeutic intervention, which holds promise for halting or reversing AD. Recent data from mouse studies show that reversing abnormal neuronal excitability in brain regions affected early in the process of Aβ deposition not only reduces Aβ load in the same brain region but also prevents the spread of Aβ pathology to other areas of the brain [16]. Since Aβ accumulation is thought to contribute to memory deficits and neurodegeneration, these results provide initial evidence that reducing neuronal excitability could provide a potential therapeutic avenue for AD. However, the exact mechanisms leading to hyperexcitability are not yet fully understood and several questions need to be answered before we can effectively advance with treatment development. In this review, we summarize the current evidence available on neuronal excitability changes in AD, highlight the gaps in the field and identify pathways forward for future research.

**Table 1:** Evidence of neuronal hyperexcitability in patients, animal and cell models of Alzheimer’s disease.

Study type/ Neuronal subtype	Model/Group of study	Functional phenotype	Methodology	Reference
**Human studies**				
Clinical study	﻿16 non-demented *APOE4* carriers (two *APOE4/4* and 14 *APOE3/4* subjects)	﻿Increased activity of parahippocampal, left hippocampal, parietal, temporal, and prefrontal regions of APOE4 carriers	fMRI	[18]
	14 non-demented *APOE3/*3 individuals			
Clinical study	Ten controls	﻿Increased hippocampal activity in MCI individuals compared to controls	fMRI	[9]
	Nine mild MCI patients	Decreased hippocampal and entorhinal activity in AD patients compared to controls		
	Ten AD patients			
Clinical study	﻿90 controls	Increased activity of the hippocampus, frontal, and temporal lobes of asymptomatic offspring of AD patients	fMRI	[17]
	95 asymptomatic offspring of AD patients			
Clinical study	15 controls	﻿Increased hippocampal activity in less impaired MCI subjects compared to controls	fMRI	[8]
	15 less impaired MCI patients	Decreased hippocampal activity in more impaired MCI and mild AD subjects compared to control		
	12 more impaired MCI patients			
	Ten mild AD patients			
Clinical study	19 controls	Increased activity in the posterior hippocampal, parahippocampal and fusiform regions of MCI patients vs controls	fMRI	[10]
	14 subjects with MCI	Brain activity of AD patients was not significantly different from control		
	11 patients with mild AD			
**Animal models**				
﻿Frontal, central, parietal, and occipital cortices of freely moving control and *hAPP-J20* mice	3–7 months non-transgenic mice (*n* = not specified)	﻿ *hAPP-J20* mice had frequent generalized cortical epileptiform discharges, which were absent in the control	EEG	[26]
	﻿3–7 months mice expressing *hAPP*			
	﻿Swedish and Indiana mutations (*n* = 6 mice)			
Neurons of L2/3 frontal cortex of live control and APP23xPS45 mice	6–10 months control mice (*n* = 10 mice; 564 neurons)	Greater number of hyperactive (>4 transients/min) neurons in *APP*^*swe*^*/PS1*^*G384*^ mice	Calcium imaging	[11]
	6–10 months *APP*^*swe*^*/PS1*^*G384A*^ mice (*n* = 20 mice; 564 neurons)			
L2/3 pyramidal neurons of cortical slices	3–4.5 months non-transgenic mice (*n* = 10 mice; 6 neurons)	Current injection induced action potential firing in APdE9 mice at subthreshold stimulus compared to controls	Whole-cell patch-clamp	[15]
Frontal cortex of freely moving control and APdE9 mice	3–4.5 months mice harboring *APP*^*swe*^ and *PSEN1*^dE9^ mutations	25–65% of APdE9 mice had seizures, while none of the control animals exhibited this phenotype	EEG	
	(*n* = 20 mice; nine neurons)			
Pyramidal neurons of lateral amygdala slices of *APOE3* and *APOE4* mice	1 and 7 months human *APOE3/E3* knock-in mice (*n* = 11 mice; 23 neurons)	﻿Reduced frequency of spontaneous excitatory postsynaptic currents in *APOE4/E4* mice	Whole-cell patch-clamp	[32]
	1 and 7 months human *APOE4/E4* knock-in mice (*n* = 12 mice; 28 neurons)			
CA1 pyramidal neurons of live control and APP23xPS45 mice	1–2 months control mice (*n* = 6 mice; 693 neurons)	Greater number of hyperactive (>20 transients/min) neurons in APP^swe^/PS1^G384A^ mice	Calcium imaging	[28]
	1–2 months *APP*^*swe*^*/PS1*^*G384A*^ mice (*n* = 7 mice; 818 neurons)			
	6–7 months control mice (*n* = 5 mice; 312 neurons)			
	6–7 months *APP*^*swe*^*/PS1*^*G384A*^ mice (*n* = 5 mice; 349 neurons)			
Neurons of CA1 hippocampal slices of APP/PS1 mice	10–14 months control mice (*n* = 16 mice; 35–75 neurons)	Higher frequency of spontaneous action potential in APP^swe^/PS1^M146V^ animals	﻿Whole-cell patch-clamp	[12]
	10–14 months *APP*^*swe*^*/PS1*^*M146V*^ mice (*n* = 16 mice; 44–69 neurons)			
Cortex and hippocampus of live control and Tg2576 mice	5wo WT mice (*n* = 17)	Synchronized transient spike-like events were detected in Tg2576 mice but absent in WT mice	EEG	[29]
	5wo *APP*^*swe*^ mice (*n* = 9)			
Neurons of L2/3 frontal cortex of live control and APP23xPS45 mice	10–14 months control mice (*n* = 9 mice; 226 neurons)	Greater number of hyperactive (>4 transients/min) neurons in APP^swe^/PS1^G384A^	Calcium imaging	[27]
	10–14 months *APP*^*swe*^*/PS1*^*G384A*^ (*n* = 10 mice; 260 neurons)			
**2D and 3D cell culture models**				
iPSC-derived neurons	﻿iPSCs from one sporadic AD patient	﻿Sporadic AD neurons had spontaneous Ca^2+^ responses and control neurons remained inactive in the absence of stimulus	Calcium imaging	[13]
﻿(*n* > 30 from three independent experiments)	iPSCs from one healthy individual			
﻿iPSC-derived neurons	﻿*APOE4/4* iPSCs from one sporadic AD patient	﻿Higher frequency of miniature excitatory postsynaptic current in *APOE4/4* neurons	Whole-cell patch-clamp	[37]
(﻿*n* = 7–9 from three independent cultures)	*APOE3/3* isogenic control iPSCs			
3D coculture of iPSC-derived neurons and astrocytes (*n* = >3 independent experiments)	﻿Lentiviral-transduced human neural progenitor cells expressing both	Increased spontaneous Ca^2+^ transients in FAD neurons	Calcium imaging	[38]
	﻿Swedish and London *APP*			
	mutations			
	Control human neural progenitor cells			
2D and 3D cultures of cortical neurons	iPSCs from one healthy individual	﻿Higher frequency of spontaneous action potential in 2D AD neurons	Whole-cell patch-clamp	[14]
(*n* = 13)	iPSCs from healthy individuals edited by CRISPR/Cas9 to express	Increase in spontaneous action potential firing rate in 3D AD neurons	MEA	
	*APP*^*swe*^ or *PS1*^*M146V*^ mutation			
	iPSCs from another healthy individual			
	iPSCs from healthy individuals edited by TALEN to express *PS1*^*dE9*^ mutation			

*AD* Alzheimer’s disease, *EEG* electroencephalogram, *FAD* familial Alzheimer’s disease, *fMRI* functional magnetic resonance imaging, *iPSC* induced pluripotent stem cells, *MCI* mild cognitive impairment, *MEA* microelectrode array, *WT* wild-type.

## Evidence of neuronal network hyperactivity from human studies

A plethora of clinical studies shows hyperactivity of the cortical and hippocampal brain areas in patients with both sporadic and familial forms of AD [8–10, 17, 18] This hyperactive phenotype is observable at the earlier stages of the disease before there is pronounced cell loss and hypoactivity [17, 19]. This is important because it could allow for early identification and intervention.

Neuronal hyperactivity has been observed in patients with mild cognitive impairment (MCI), a state between the expected cognitive changes during aging and dementia. Individuals suffering from MCI have a greater risk of developing AD, but MCI does not always progress to dementia [20]. A number of clinical studies have used functional magnetic resonance imaging (fMRI) to measure changes in blood oxygenation as a proxy for brain activity. While performing associative memory-encoding tasks, patients with MCI displayed increased brain activity in the hippocampal, parahippocampal, and fusiform regions. The AD group, on the other hand, showed hippocampal and entorhinal hypoactivation [8, 9], although one study found no difference between AD and control [10]. Together these findings suggest neuronal hyperactivity precedes hypoactivity in AD. An interesting study by Basset et al. [17] investigated the neuronal activity of non-symptomatic children of autopsy-confirmed AD patients with at least one more first-degree relative clinically diagnosed with AD. The fMRI scans acquired during an associative learning task showed greater activity in the frontal and temporal lobes, including the hippocampus, compared to age-matched controls without any history of first-degree relatives with suspected dementia. Their results demonstrated that individuals at genetic risk of developing LOAD showed increased brain activity, starting as early as a decade before the onset age of their parents’ disease.

It is important to note that excessive neuronal network activation can also manifest as epilepsy. A meta-analysis of 30 studies concluded that AD patients have an increased risk of suffering from epilepsy and seizures [21]. The risk for epileptic seizures is particularly higher in patients with younger-onset AD and is greater during earlier stages of the disease [22, 23]. Together, these results suggest brain hyperactivity occurs early in the development of AD. Since imaging studies examining living patients are unable to provide us with the resolution necessary to observe pathology at the cellular and molecular levels, we turn to animal and cell models of the disease for this purpose.

## Animal models examining hyperexcitability

A variety of mouse models to study AD has been generated by genetically modifying animals to express AD-related mutations, most commonly in the *APP* and *PSEN1* genes. Less commonly, but still of great importance, are mouse models expressing the human *APOE4* allele, the main genetic risk factor for LOAD [24]. Mouse models of AD develop several of the main hallmarks of the disease, such as amyloid plaques and/or tau pathology and cognitive impairment [25], providing a model system that can be manipulated to identify pathological mechanisms and test potential treatments.

Aberrant neuronal excitability has been reported in the frontal cortex and hippocampus of several animal models of AD [11, 12, 26–28]. For example, by measuring cortical and hippocampal electrical activity with electroencephalogram (EEG) recordings, Palop et al. [26] detected spontaneous non-convulsive seizure activity in hAPPFAD mice, which were absent in controls. Busche et al. [11] used two-photon Ca^2+^ imaging to analyse brain activity in real-time in vivo by measuring intracellular Ca^2+^ transients of individual neurons of layer 2/3 frontal cortex. In double transgenic 1.5 to 2-month-old APP23xPS45 mice, no significant differences in Ca^2+^ transients were found between AD and control mice. At this age, no memory deficits were detected, and amyloid plaques were absent, as they start to form at the age of 3 months. However, at an older age (6 to 10-month-old), the number of silent neurons was increased in plaque-bearing APP23xPS45 mice, and the proportion of hyperactive cells was 15 times higher than in wild-type (WT) animals. At this age, spatial and working memory was impaired in AD mice. Furthermore, at older ages, 10 to 14-month-old APP23xPS45 mice had more hyperactive neurons than WT [27]. Compared to 6 to 8-month-old mice, both WT and APP23xPS45 (10 to 14-month-old) mice had an increase in hyperactive neurons, but only APP23xPS45 mice had fewer silent neurons [27]. This suggests hyperactivity occurs in both healthy aging and AD but is more pronounced in the AD phenotype/model. An important observation from these studies is that the animal model used did not show the shift from hyper to hypoexcitability in the cortex that is reported in patient studies [8, 9]. Hence, the use of this particular animal model to study hyperexcitability in AD may be performed with caution and the results should be corroborated by other models.

Curiously, in the cortex, the hyperactive neurons were localized near the amyloid plaques (<60 µm), whereas silent and normal cells were randomly distributed [11]. This same pattern was observed in the hippocampus of APP23xPS45 mice [28], though with an earlier presentation of the hyperactivity. CA1 pyramidal neurons of the hippocampus had a greater number of hyperactive neurons compared to WT by 1 to 2 months of age. At an older age (6 to 8-month-old), AD mice had increased hyper- and hypoactive neurons. The number of hyperactive neurons, however, was significantly reduced compared to younger mice [28]. These results suggest that, in the hippocampus, hyperactivity starts before there is marked neuronal silencing and decreases with age, while hypoactivity increases over time, which is in line with clinical findings [8, 9]. These studies also indicate that functional neuronal deficits start earlier in the hippocampus and eventually progress to the cortex. It is relevant to note that most animal models used to study AD express more than one AD-related mutation, which differs from the clinic, as FAD patients harbor only single mutations. However, studies using animal models expressing a single AD-related mutation have also detected neuronal hyperexcitability [29–31].

In the amygdala, analysis of single neurons by patch-clamp revealed contrasting results compared to the hippocampus and cortex. The flow of ions that depolarizes postsynaptic neurons and renders them more likely to fire an action potential is termed the excitatory postsynaptic current. Reduced spontaneous excitatory postsynaptic current frequency was found in 1 and 7-month-old mice bearing the human *APOE4* gene, compared to mice with the human *APOE3* gene, with no differences in inhibitory currents [32, 33]. Aged *APOE4* mice (18 to 20-month-old mice) demonstrated an increased frequency of both excitatory and inhibitory transmission, with greater inhibition overall [34]. This was observed in the absence of Aβ plaque deposition or NFT formation.

Taken together, these results suggest that neuronal hyperactivity occurs in distinct brain regions of animal models of AD and manifests at different stages of the disease, depending on the area of the brain. These brain region differences might be explained by differences in neuronal vulnerability to excitability changes. Selective vulnerability is common in neurodegenerative diseases and may explain why some brain regions are affected earlier during the disease course, while others remain intact. Hence, some neuronal subtypes may be more susceptible to the different pathways underlying hyperexcitability, and therefore would be the first to show such alterations. As the disease progresses, this pathological change spreads to less vulnerable areas of the brain, while others may be unaffected. More studies are necessary to confirm the exact timeline in which hyperexcitability occurs and which brain regions are affected. Then, more efforts can be directed at identifying the precise neuronal populations that are most vulnerable and understanding what makes them vulnerable. Targeting proteins or receptors exclusively expressed in those neuronal subtypes would allow us to specifically target these neurons to reduce neuronal hyperexcitability.

## Cell models of neuronal hyperexcitability

Induced pluripotent stem cells (iPSCs) are beneficial in the study of human neurological disorders that are caused by a complex range of genetic risk factors, including AD. One strength of iPSC disease modeling is that the cells maintain the genetic background of patients and can be used to identify how specific genes lead to pathology at the cellular and molecular levels. Pluripotent stem cells can be differentiated into neurons in two-dimensional cultures; however, the lack of Aβ aggregates due to regular cell media change is one of the disadvantages that has been documented by some studies in this model [35]. This issue may be addressed to a certain extent with 3D neuronal cultures/organoids, which can recapitulate amyloid-β aggregation, as well as NFT pathology [36].

A handful of studies have looked into neuronal excitability in LOAD using cell models. LOAD iPSC-derived neurons showed an increase in spontaneous Ca^2+^ signals, compared to controls that were silent in the absence of stimulus [13]. In another study, iPSC-derived neurons from a LOAD patient harboring the *APOE4* allele displayed higher excitatory current frequencies compared to *APOE3* isogenic control neurons [37]. A thorough study by Ghatak et al. found that iPSC-derived neurons bearing either *PSEN1*^dE9^, *PSEN1*^M146V^ or *APP*^swe^ mutations were hyperactive, compared to their isogenic controls [14]. Organoid models of AD, which displayed cortical layer formation and ﻿Aβ accumulation, also showed an enhanced extracellular action potential firing rate, detected with microelectrode arrays [14]. Another 3D cell model using neural stem cells genetically modified to express the K670N/M671L (Swedish) and V717I (London) mutations in the *APP* gene, found neurons from the AD group had increased spontaneous Ca^2+^ transients, compared to controls [38]. The proportion of hyperactive neurons increased over time in culture, with just over 15% of neurons showing hyperactivity at 3-weeks and more than 65% at 9-weeks of differentiation. Animal and human studies suggest that later stages of the disease lead to reduced neuronal activation [9, 39, 40]. Thus, it would be interesting to verify whether the number of hyperactive neurons would continue to increase or if it would decrease, in line with in vivo observations.

In summary, all 2D and 3D cell models of AD harboring different FAD mutations or derived from LOAD patients appear to show a similar hyperexcitable phenotype, corroborating findings from human and animal studies. These results suggest that hyperexcitability is detected across different systems used to model and study AD.

## Potential causes of neuronal hyperexcitability

**Fig. 1:**
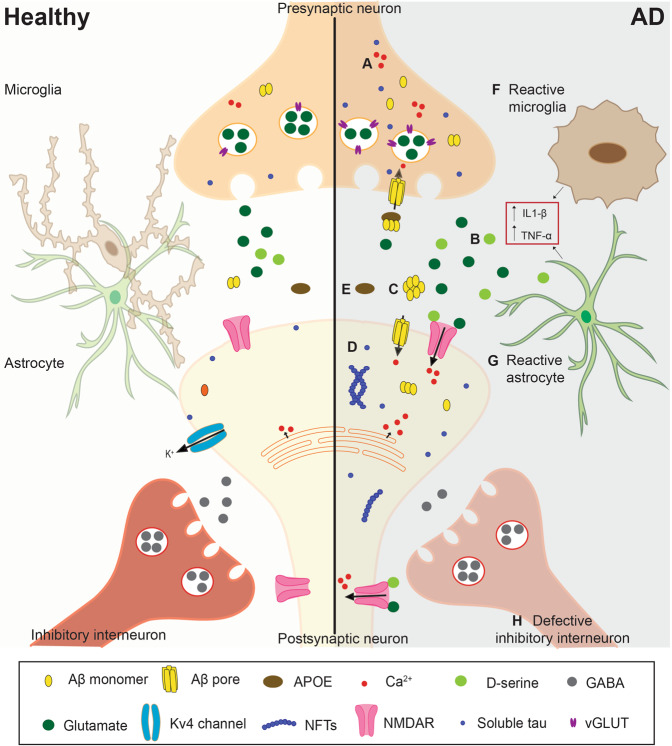
Mechanisms causing neuronal hyperexcitability in Alzheimer’s disease. Representation of a synapse in the healthy (left) and in the AD (right) brain. **A** Increased release of calcium from intracellular stores from pre and postsynaptic neurons results in higher levels of cytosolic calcium. **B** Enhanced glutamatergic signaling can be caused by reduced astrocytic uptake, reduced levels of glutamine synthetase (not shown in the Figure), and/or increased vGLUT expression. **C** Amyloid-β can form ionic pores in the plasma membrane. It also reduces the expression of Kv4 channels and increases NMDAR activation via increased d-serine and glutamate release and reduced glutamate uptake. **D** Protein tau can contribute to hyperexcitability by altering glutamate levels as well as the expression and function of Kv4.2 channels and NMDAR. **E** Compared to apoE3, apoE4 reduces the clearance and uptake of Aβ42 by astrocytes and microglia, respectively. **F**, **G** The release of pro-inflammatory cytokines, such as IL-1β and TNF-α, by glial cells can promote hyperexcitability. **F** During gliosis, reactive microglia fail to properly regulate neuronal excitability. **G** In AD, astrocytes show increased release and reduced uptake of glutamate, as well as reduced expression of potassium channel Kir4.1 (not shown in the Figure). Reactive astrocytes also increase neuronal excitability by reducing synaptic inhibition. **H** Reduced firing frequency and the number of inhibitory GABAergic neurons is another contributing factor to hyperexcitability in AD. AD Alzheimer’s disease, NMDAR NMDA receptor, vGLUT vesicular glutamate transporter.

Hyperexcitability is observed in LOAD and FAD patients, as well as various laboratory models of the disease. Because AD is a multifactorial disease it is likely that several pathways contribute to hyperexcitability. In this section, we present the evidence pertaining to a range of factors that have been proposed to be involved in hyperexcitability: Ca^2+^ dyshomeostasis; glutamate and *N*-methyl-d-aspartate receptors (NMDAR); amyloid-β; tau; genetic risk factors for LOAD, glial cells, and inhibitory interneurons (Fig. 1).

## Ca^2+^ dyshomeostasis

Mutations in the presenilin genes, *PSEN1* and *PSEN2*, that are part of the γ-secretase complex, can cause FAD [5]. Dysregulation of intracellular calcium levels via mutations in these genes has been reported to contribute to AD pathology [41]. Apart from their role in the processing of amyloid precursor protein (APP), the presenilins also play a role in regulating Ca^2+^ levels in the cytoplasm by acting on different receptors in the endoplasmic reticulum (ER) membrane [42–45]. Knocking out *PSEN1* and *PSEN2* or expressing a FAD mutation in mouse hippocampal neurons increased ER Ca^2+^ levels and consequently elevated Ca^2+^ release into the cytoplasm in the presence of a stimulus [42]. Presenilins have been suggested to function as Ca^2+^ leak channels in the ER, which become defective with *PSEN* mutations [43]. Alternative mechanisms of how presenilin disturbs Ca^2+^ signaling have also been proposed. For example, the sarcoendoplasmic reticulum Ca^2+^ ATPase (SERCA) pump maintains the low cytosolic levels of Ca^2+^ by pumping this cation from the cytosol into the ER. Knocking out both *PSEN1* and *PSEN2* genes in fibroblasts reduced the activity of SERCA and consequently, ER Ca^2+^ stores, and increased cytosolic Ca^2+^ [44]. Finally, the inositol trisphosphate receptor (InsP3R), an ER Ca^2+^ channel that releases Ca^2+^ into the cytoplasm, is also affected by presenilin-1 alterations. Mutations in *PSEN1* enhanced InsP3R gating, elevating cytosolic Ca^2+^ levels [45]. Additionally, emptying presynaptic Ca^2+^ stores reduced amplitude and frequency of presynaptic Ca^2+^ transients and normalized neuronal network activity in both APP^swe^/PS1^G384A^ and PS45 mice [27]. Since intracellular levels of Ca^2+^ mediate neurotransmitter release, elevated cytosolic Ca^2+^ can trigger excitatory neurotransmitter release and lead to exacerbated neuronal excitability.

## Glutamate and NMDA receptors

Excessive glutamate is toxic for cells and has been associated with neurodegeneration [46]. As the main excitatory neurotransmitter in the central nervous system, glutamate must be maintained at appropriate levels to promote cell survival [47]. At high levels, glutamate causes excitotoxicity and neuronal death [48]. To prevent overstimulation of the receptors, after glutamate is released in the synapse, it is taken up by astrocytes through the excitatory amino acid transporters (EAAT). Reduced glutamate transport has been detected in the cortex and hippocampus of AD patients, which some studies reported to be due to a reduction in the expression of EAAT1 and EAAT2 [49–51]. When taken up by astrocytes, glutamate is converted into glutamine by glutamine synthetase. Glutamine is then transferred back to the neurons where it is recycled into glutamate and transported into synaptic vesicles and becomes available to be released again. In the frontal and temporal cortices of postmortem brain tissue of AD patients, there is a reduction in the levels of glutamine synthetase, thus increasing the availability of glutamate [52–54]. The release of glutamate in the synapse is dependent on the import of glutamate into synaptic vesicles by vesicular glutamate transporters (vGLUT). An iPSC-derived neuronal culture found increased expression of vGLUT1 in the AD group, which could lead to higher release probability [14]. Another study, however, found reduced levels of vGLUT in hippocampal neurons of *APOE4* mice [55]. As for cerebrospinal fluid (CSF) levels of glutamate in AD patients, results so far have been controversial. While some authors have reported higher levels of glutamate [56, 57], others observed no changes [58] or even reductions in glutamate levels [59, 60]. Differences in the duration of illness, history of medication, and the inclusion or not of biomarkers as criteria for the diagnosis of AD could explain such variability in the results.

There are three types of ionotropic glutamatergic receptors and of these, NMDARs have been implicated in neuronal hyperexcitability in AD [27, 61] Application of ﻿ the activity-dependent NMDAR blocker MK-801 in AD mice reduced the frequency of neuronal firing in normal and hyperactive neurons, suggesting aberrant activation of this receptor is involved in hyperexcitability [27]. The use of the drug Memantine, which preferentially blocks extrasynaptic NMDAR, to treat moderate to severe AD in the clinic, supports the theory that synaptic NMDAR are required for neuronal survival, while extrasynaptic NMDAR mediate neurotoxicity [62]. Consistent with this hypothesis, enhancing the synaptic glutamate NMDAR subunit epsilon-1 (GluN2A) with the positive allosteric modulator, GNE-0723, reduced network hypersynchrony and epileptic discharge and ameliorated memory deficit in an AD mouse model [61]. The authors speculated this could be a consequence of activation of GluN2A expressing interneurons, thus suppressing excitatory neuronal activity. However, further investigation is required to fully understand the mechanisms behind these results. In conclusion, several alterations in glutamatergic transmission are observed in AD, thus modulation of specific NMDAR subunits poses a potential treatment strategy for hyperexcitability in AD.

## Amyloid-β

Amyloid-β is one of the main components of amyloid plaques, insoluble extracellular deposits found in the brain of AD patients. The presence of amyloid plaques in postmortem brain tissue is one of the criteria for a definitive diagnosis of AD. Importantly, ﻿increased neuronal excitability is observed in neurons in the vicinity of Aβ plaques [11, 28] and a few reports have shown both soluble and fibrillar forms of Aβ can provoke changes in neuronal activity. Administration of Aβ dimers directly to neurons increased action potential firing, meanwhile, γ-secretase inhibitors, which reduced the levels of soluble Aβ, also restored neuronal activity to control levels in mice and in cell culture [14, 28]. In *APP*^dE9^ mice, administration of fibrillary but not oligomeric Aβ depolarized both dentate gyrus and layer 2/3 pyramidal neurons and enhanced their activity. The authors proposed the insertion of fibrillar Aβ in the membrane could lead to the disruption of voltage-gated ion channels responsible for maintaining neuronal membrane potential [15]. Supporting this theory, a *Drosophila* model expressing human Aβ42 found a role for Kv4 channels in Aβ-induced hyperexcitability. Neurons overexpressing Aβ42 were hyperexcitable and expressed less Kv4 protein, while Kv2 and Kv3 channels were unaffected. Increasing Kv4 channel levels in the animals not only restored normal neuronal excitability but also rescued learning impairment [63]. Aβ can also perturb Ca^2+^ homeostasis by stimulating voltage-gated calcium channels or directly by forming ion-permeable pores in the plasma membrane and increasing Ca^2+^ influx [64, 65]. Another possible mechanism through which Aβ could participate in neuronal hyperactivity is by modulating glutamatergic signaling. In cultured cells, Aβ42 augmented the release of the NMDAR co-agonist d-serine by microglia and glutamate from astrocytes [66, 67]. Soluble Aβ oligomers decreased the uptake of glutamate and increased extrasynaptic NMDAR activation, which is probably due to a spillover of excessive extracellular glutamate [68–70]. Moreover, the application of soluble Aβ in the CA1 region of the hippocampus in WT mice increased neuronal activity by blocking glutamate reuptake [71]. Studies have also shown neuronal hyperactivity can promote Aβ accumulation in mice, therefore creating a vicious cycle that is detrimental to the cells [72, 73]. In sum, accumulation of Aβ affects numerous pathways that converge on a similar outcome, an increase in neuronal excitability.

## Tau

Alzheimer’s disease can be classified as a tauopathy, a group of neurodegenerative diseases characterized by the aggregation of hyperphosphorylated tau proteins into neurofibrillary tangles in neurons [74]. Tau is a multifunctional protein, initially identified as a microtubule-binding protein. Mutations in the gene encoding tau (*MAPT*) can cause frontotemporal dementia, another tauopathy that is also associated with increased excitability of neuronal networks [75]. In mice and *Drosophila* models of epilepsy, reduction or ablation of endogenous tau expression improves cortical and hippocampal neuronal hyperexcitability and reduces the incidence and severity of seizures [76–79]. In transgenic mice, overexpression of the human tau protein harboring the A152T mutation increased levels of extracellular glutamate and neuronal loss in vivo [80]. A mouse model of tauopathy, expressing human tau with a P301L mutation, displayed increased neuronal excitability in layer 3 of the cortex even before the formation of NFT, which could indicate a role of soluble hyperphosphorylated tau in driving this aberrant phenotype [81]. In the same mouse model it was also observed that while pyramidal neurons in the CA1 region of the hippocampus had increased firing, inhibitory interneurons were less active, suggesting a failure in inhibitory synaptic transmission [82]. In animal models of AD specifically, tau reduction was also protective against hyperexcitability. Transgenic hAPP mice expressing tau had heightened sensitivity to the γ-aminobutyric acid (GABA) receptor antagonist, pentylenetetrazole, and the glutamate receptor agonist, kainate, suffering from seizures at lower doses compared to control mice. Tau reduction blocked this effect and reversed memory deficits without changing Aβ levels [83]. More precisely, Roberson et al. [78] found that decreased tau levels reversed NMDAR dysfunction and the imbalance of inhibitory and excitatory currents that shifted towards excitation in hAPP-tau^+/+^ mice. Similar to Aβ, the release and propagation of tau is stimulated by neuronal activity, generating a toxic vicious cycle that could accelerate the progression of AD [84, 85]. Looking deeper into the pathways modulated by tau, Hall et al. [86] reported that knocking out tau in AD mice rescued dendritic hyperexcitability and Kv4.2 potassium channel depletion in the CA1 region of the hippocampus. Knocking out Kv4.2 channels in mice contributed to behavioral abnormalities and elevated epileptiform spiking. Interestingly, EEG recordings of frontal and parietal cortices of transgenic mice detected increased epileptiform spikes in hTau-A152T mice but decreased in hTau-WT mice compared to controls [87], indicating that overexpression of tau in animal models can lead to different outcomes, depending on the presence or absence of a disease-causing mutation.

Recent work indicates tau may also drive hypoexcitability. Transgenic mice expressing human tau with the P301L mutation had reduced neuronal activity compared to the control [28, 88]. Furthermore, the reduction in neuronal activity appears to be driven by soluble tau as this phenotype was observed in the absence of NFT [28, 89]. It is important to note, however, that mutations in the *MAPT* gene cause frontotemporal dementia but have not been linked to AD directly, therefore the interpretation of these results in the context of AD must be taken with caution. Using mouse models of AD, studies have shown that while Aβ pathology increases excitability, tau pathology drives hypoexcitability, and when both pathologies are combined, the effect of tau seems to predominate, leading to decreased neuronal firing [90, 91]. A proposed mechanism through which tau could lead to neuronal hypoexcitability involves the axon initial segment (AIS), where the initiation of the action potential occurs. Hatch et al. [88] found tau hyperphosphorylation destabilizes microtubules, shifting the AIS further away from the soma and consequently reducing neuronal firing frequency. Another interesting finding by the authors was that phosphorylation of different tau residues is commonly observed in AD (identified using the antibodies AT180 (T231), 12E8 (S262/S356), and PHF1 (S396/404), led to different outcomes. While AT180E and 12E8E hyperphosphorylation in primary hippocampal neurons caused the relocation of the AIS further from the soma, PHF1 had no effects on the location of the AIS.

Taken together, these results indicate the effects of tau can vary depending on phosphorylation, mutation, and brain region, thus deeper investigation into the role of tau in mediating neuronal excitability changes is required to understand its impact on AD.

## *APOE4* and genetic risk factors for LOAD

Sporadic AD or LOAD comprises more than 98% of all cases of AD [6]. Whilst LOAD is not caused by a mutation in a single gene, there is a significant genetic component, with multiple genetic variants affecting the risk of developing LOAD. The *APOE* ε4 allele is the major genetic risk factor for LOAD, elevating the risk of developing the disease 3 to 10-fold, compared to the general population [92]. The human *APOE* gene encodes three isoforms of the apolipoprotein E (apoE): *APOE* ε2, *APOE* ε3, and *APOE* ε4. The protein apoE is involved in the transport of cholesterol and other lipids in the periphery and in the brain [93]. Increasing evidence shows hyperexcitability in humans and animal models harboring the *APOE4* allele, indicating this phenotype is not limited to the familial form of the disease. In humans, young and healthy individuals carrying the *APOE4* allele had greater hippocampal activation at rest and during a memory-encoding task relative to non-*APOE4* carriers. This was observed along with a lack of difference in brain perfusion or whole-brain, gray matter, white matter, cerebrospinal fluid (CSF) or hippocampal volumes [19]. ﻿Increased brain activity was also observed in the prefrontal, temporal and parietal cortices of older, cognitively-intact *APOE4* carriers [18, 94–96]. Mice expressing human *APOE4* developed a seizure phenotype that is absent (or less pronounced) in mice expressing human *APOE2* or *APOE3*. *APOE4* mice showed increased hippocampal and cortical activity and were also more sensitive to the GABA receptor antagonist, pentylenetetrazole. Interestingly, these alterations were observed in the absence of Aβ plaque formation, with no significant changes in the levels of Aβ42, although Aβ40 was increased in *APOE4* animals [97]. Apart from enhanced excitability, *APOE4* mice have impaired short-term synaptic plasticity [98]. Moreover, *Apoe* knock-out mice displayed reduced α-amino-3-hydroxy-5-methyl-4-isoxazolepropionic acid (AMPA)/NMDA receptor ratios, while mice lacking *Apoe* expression only in the brain displayed similar NMDAR and AMPAR currents to *APOE3* mice. This suggests plasma apoE plays an important role in synaptic function [99].

Studies have identified defective dendritic morphology in *APOE4* mice, including reduced dendritic length, branch number, spine size, and spine density [98, 100, 101]. This could be explained by a deficiency in the ability of the apoE4 isoform to deliver lipids, such as cholesterol. Cholesterol and other lipids are important for neuronal morphology, synapse formation, and ion channel function, thus alterations in their levels can dramatically affect the excitability of neurons [33]. In culture, *APOE4* human iPSC-derived neurons are more excitable than *APOE3* isogenic controls, which could be due to an increase in the expression of synaptic proteins, including synaptophysin and PSD-95, and an upregulation of genes involved in neuronal differentiation [37]. The *APOE* genotype also has an effect on the levels of Aβ. Compared to *APOE3*-expressing cells, iPSC-derived astrocytes and microglia carrying the *APOE4* allele have reduced clearance and slower uptake of Aβ42, respectively [37]. However, Konttinen et al. [102] found there was no significant difference in Aβ42 uptake in *APOE4* iPSC microglia, compared to *APOE3* isogenic control lines. In regards to the difference between *APOE2* and *APOE3* astrocytes, Brookhouser et al. [103] observed that the effect of the *APOE* genotype in Aβ uptake in iPSC-derived astrocytes varies depending on the AD-related mutation harbored by the cell. Thus, further research is required to understand neuronal excitability changes and the complex interaction between microglia, astrocytes and neurons under different *APOE* genotypes. This could be accomplished by coculturing these cell types with different combinations of *APOE* alleles.

Neuroinflammation is a common feature of AD [104], and genome-wide association studies (GWAS) have identified several genes involved in the immune response to be associated with AD [105, 106]. Some of these genes are *CR1, CD33, MS4A*, *TREM2*, *CLU, ABCA7, EPHA1*, and *BIN1*. In the context of the immune response, *BIN1* knock-out mice display a higher incidence of inflammation [107]; however, this gene is also involved in endocytosis, calcium homeostasis, and apoptosis [106]. *BIN1* encodes the bridging integrator 1 (BIN1), myc box-dependent-interacting protein, which is expressed throughout the body with higher levels in muscle and brain tissue [108]. Little is known about BIN1 function in the nervous system, however, it has recently been implicated in neuronal hyperexcitability. In rat hippocampal neuronal cultures, overexpression of *BIN1* increased calcium influx and action potential firing frequency due to an increase in the interaction between BIN1 and ﻿L-type voltage-gated calcium channels. The interaction between these proteins is tau dependent, since reducing tau expression inhibited their interaction and consequently, reversed this hyperexcitable phenotype [109].

In summary, numerous studies support the role of *APOE4* in hyperexcitability and more recent data has revealed *BIN1* to be another potential contributing factor to AD neuronal excitability changes. GWAS offers the first step in elucidating the genetic risk factors for developing AD. From there, candidate genes can be manipulated in laboratory models to clarify which pathways are involved in neuronal hyperexcitability.

## Glial cells and neuroinflammation

Glia are non-neuronal cells of the central nervous system, including astrocytes, microglia, and oligodendrocytes. The latter are responsible for the formation of myelin sheaths around axons [110], and even though oligodendrocyte abnormalities have been reported in AD (see refs. [111, 112] for more on oligodendrocytes in AD), in this review we will focus on the role of astrocytes and microglia. Astrocytes and microglia are activated by, and respond to, pathogens and stressors by releasing pro-inflammatory molecules to promote repair; however, chronic activation of glial cells and inflammation can promote seizures [113], which are more prevalent in AD patients than in healthy elderly people [21]. Injection of the pro-inflammatory cytokine, interleukin-1 β (IL-1β), exacerbates seizures [114], while blockade of the IL-1β receptor reduces seizure susceptibility in mice [115, 116]. Inhibition of IL-1β synthesis also shows anticonvulsive effects [117]. The pro-convulsive effect of IL-1β is reversed by treatment with an NR2B receptor antagonist, indicating the involvement of the NMDAR in this pathway [118]. The pro-inflammatory cytokine, tumor necrosis factor-α (TNF-α), regulates glutamatergic signaling in both neurons and astrocytes. Exposure of cultured hippocampal neurons to TNF-α increased the expression of AMPA receptors in the plasma membrane, which resulted in a higher frequency of miniature excitatory postsynaptic currents [119]. In the dentate gyrus of mice, TNF-α is required for glutamate release from astrocytes, causing an increase in excitatory neuronal activity via activation of NMDA receptors [120]. Reduction of neuroinflammation, including anti-TNF-α therapy, has been shown to protect against epilepsy in patients and animals [121, 122]. Therefore, this approach to reduce neuroinflammation and consequently hyperexcitability could also be relevant in the context of AD.

### Microglia

Genes involved in the immune response have been linked to a higher risk of developing AD [106]. Many of these genes are highly expressed in microglia, such as *ABCA7*, *CD33*, and *TREM2. TREM2* encodes triggering receptors expressed on myeloid cells 2 (TREM2), a protein that stimulates microglial phagocytosis [123]. Deletion of *TREM2* enhances tau and amyloid-β pathology [124, 125], suggesting a direct role for microglia in containing the spread of abnormal protein aggregation in AD. Microglia are the brain’s resident macrophages, playing an important function in brain injury and inflammation, as well as synaptic pruning [126]. Apart from these functions, microglia can also sense and modulate neuronal activity [127]. Similar to neurons, microglia residing close to amyloid plaques show increased Ca^2+^ transients [128], which can occur as a response to neuronal hyperactivity [129]. In zebrafish, resting microglia sense neuronal activity and extend processes towards these neurons. This interaction, in turn, reduces both spontaneous and evoked neuronal activity [130]. A similar outcome is observed in mice, where the increased neuronal firing was followed by a greater number of microglial extensions contacting the active neurons, and pharmacological inhibition of microglia exacerbated neuronal responses to neurostimulants [131]. Microglial depletion also aggravated seizure severity in mice [132]. However, microglia-neuronal contact can also result in increased synaptic activity and network synchronization [133]. In mice, Ca^2+^ responses of motor cortical neurons were elevated in dendritic spines in close contact with microglia. This enhanced response was reduced after the retraction of microglial processes. Ablation of microglia reduced the synchronous firing of neurons located close to each other. These contrasting results probably highlight the function of microglia as a regulator of neuronal activity. Microglia can sense neuronal hypo- and hyperexcitability [127] and can promote either an increase or decrease in neuronal firing, which indicates these cells may be critical for maintaining homeostatic network activity in the brain. Interestingly, microglial activation with lipopolysaccharide (LPS) treatment abolished the regulation of neuronal activity, suggesting brain inflammation can have a detrimental effect in microglial modulation of neuronal circuits [133].

### Astrocytes

Astrocytes exert a range of functions in the brain, including nutrient supply to neurons, maintenance of the blood–brain barrier, and regulation of the extracellular ionic environment [134]. Astrocytes regulate ion homeostasis by clearing extracellular K^+^ during neuronal repolarization [135]. Defective K^+^ buffering by astrocytes increases extracellular K^+^ levels and is associated with the initiation of seizures [136]. Protein expression of the astrocytic potassium channel Kir4.1 is reduced in a mouse model of AD and in the brain of AD patients [137], which could impact the resting membrane potential and neuronal excitability. In addition to K^+^, astrocytes also regulate extracellular glutamate levels by taking up this neurotransmitter from the synapse via EAAT1 and EAAT2 receptors, which have reduced expression in the AD brain [49–51]. Elevated glutamate release by astrocytes was also found in a mouse model of AD [138]. Astrocytic dysregulation of glutamate levels contributes to neuronal hyperexcitability and therefore, represents a potential target in re-establishing physiological neuronal activity. Under pathological conditions, such as AD, astrocytes go through molecular and functional changes in a process known as astrogliosis or reactive astrogliosis [139]. The selective induction of astrogliosis in mice decreased synaptic inhibition of CA1 pyramidal neurons, as well as expression of glutamine synthetase [140]. At the cellular level, inhibitory postsynaptic currents were smaller in neurons neighboring reactive astrocytes, while excitatory postsynaptic currents were unaltered. These changes resulted in hyperexcitability of the neuronal network. The application of glutamine reversed these aberrant phenotypes, suggesting reactive astrocytes can impair network excitability by depleting glutamine available for neuronal uptake and reducing the availability of GABA for release by inhibitory neurons.

## Inhibitory interneurons

Inhibitory interneurons regulate neuronal network oscillations, such as gamma waves, which are required for cognitive functions [141]. Increased gamma oscillations during memory-encoding tasks are predictive of effective memory formation and are associated with reduced epileptiform discharges [142]. A loss of inhibitory neuron function has been associated with hyperexcitability in AD. Reduced inhibitory tone, probably due to decreased responsiveness to GABAergic stimuli, lower numbers of GABAergic interneurons, and reduced GABAergic signaling, has been reported in *APOE*4 mice [100, 143]. Neuronal cultures with either *PSEN1*^M146V^ or *APP*^swe^ mutation, which were hyperactive, presented reduced frequency of inhibitory postsynaptic currents, as well as decreased staining for GABA and the vesicular GABA transporter [14]. Inhibitory neuron deficiency has been reported in the cortex and hippocampus of animal models of AD. Treating APP23xPS45 mice with the GABA_A_ receptor antagonist, gabazine increased the firing frequency of hyperactive, silent, and normal neurons of cortical layer 2/3 to a similar level. The increase was, however, significantly smaller for hyperactive neurons than for normal cells [11]. EEG recordings of hAPPFAD mice showed increased spontaneous epileptiform discharges during low-intensity gamma oscillations, which were reduced in hAPPFAD animals, compared to controls [144]. Since gamma waves are generated by the activity of parvalbumin (PV) positive GABAergic interneurons, the authors tested if abnormalities in PV cells could lead to network hypersynchrony [144]. Patch-clamp confirmed reduced inhibitory postsynaptic current frequency in hAPPFAD mice. These animals also had reduced expression of the voltage-gated sodium channel Nav1.1 in the parietal cortex and PV cells had reduced mRNA and protein expression of Nav1.1. Overexpressing Nav1.1 in PV cells prevented inhibitory postsynaptic current abnormalities and reduced epileptiform discharges by decreasing the number of spikes during low-intensity gamma oscillations. Impaired Nav1.1 appears to contribute to cognitive decline, as increasing its expression improved spatial learning and memory in mice [144]. Hamm et al. [145] found similar results in the hippocampus of TgCRND8 mice, transgenic mice containing human *APP* with both K670N/M671L (Swedish) and V717F (Indiana) mutations. The animals exhibited fewer PV interneurons in the CA1, as well as depressed gamma oscillations. A decreased expression of Nav1.6 and a tendency towards a reduction for Nav1.1 was also detected. These aberrations occurred concomitantly with impaired memory performance. Another study has further corroborated the role of Nav1.1 in impaired synaptic inhibition. Nav1.1 expressing interneuron transplant in the hippocampus and cortex of hAPPFAD mice reduced epileptic spike frequency and enhanced gamma oscillatory activity. Transplantation of Nav1.1 interneurons also reversed learning deficits [146]. To sum up, these studies show a clear contribution of Nav1.1 and impaired synaptic inhibition to elevated neuronal excitability in AD. Targeting this channel in future studies has the potential for developing new treatments for AD. Even though transplantation studies have shown beneficial effects, it could still take years for this approach to be translated into the clinic. A potentially easier solution would be enhancing Nav1.1 activity with selective drugs, a strategy that has been shown effective in reducing seizure activity in animal models of Dravet syndrome epilepsy [147, 148]. The effect of these compounds in reducing hyperexcitability in AD has yet to be investigated.

### Is hyperexcitability neuroprotective or neurodegenerative?

Synaptic deficits, neurodegeneration, and brain atrophy are common features of AD [149]. Because of this, it has been suggested that neuronal hyperexcitability could be an attempt to compensate for the neuronal loss or synaptic deficits by recruiting greater neuronal resources, to maintain the same level of performance. However, neuronal hyperactivation has been documented in individuals with MCI who still present poorer memory performance compared to controls [10]. Also, previous studies have found enhanced brain activity in individuals at risk for developing AD, even with unchanged hippocampal volume [19]. This hypothesis is further refuted by evidence that antiepileptic drugs decrease hippocampal activation and improve memory performance in individuals with MCI, as well as in animal models of AD [150, 151]. Thus, increased brain activity is more likely a driver of AD pathogenesis rather than a compensatory effect. In fact, augmented brain activity can promote AD pathology. Increased neuronal activity not only causes increases in Aβ levels [72, 73] but also stimulates the release of tau in vivo and in vitro, leading to the spread of tau pathology, which contributes to cognitive deficits [84, 85]. Besides, reducing neuronal excitability decreases Aβ deposition and synaptic loss and prevents the spread of Aβ plaques in mice [16, 152].

A longitudinal study recording hippocampal activity in older individuals without dementia found people with the most pronounced hyperactivation at the first assessment suffered the greatest hypoactivation two years later and the fastest cognitive decline [39]. AD patients with subclinical epileptiform activity had faster cognitive decline than those who did not present such abnormalities [153]. Therefore, network hyperexcitability may be predictive of imminent hypoactivity and cognitive impairment [39]. A similar pattern of neuronal excitability dysregulation is observed in another neurodegenerative disease, amyotrophic lateral sclerosis (ALS; reviewed in ref. [154]). Motor neuron hyperexcitability is a common pathology observed in both sporadic and familial cases of ALS. Hyperexcitability occurs in the early stages of ALS, even prior to motor symptom onset, and then progresses to hypoactivity [154]. It has been shown that the neurons most vulnerable to degeneration in ALS are also the ones that receive greater excitatory input, compared to the resistant neuronal subtypes, and eliminating this input protects against degeneration [155]. Whether the same process occurs in AD has yet to be identified.

Together, current evidence suggests that hyperactivation occurs in the early stages of AD, even before there is significant neuronal loss, contradicting the hypothesis that overexcitation is a compensatory effect. On the contrary, neuronal hyperactivity appears to be detrimental by promoting AD pathology and possibly, cognitive decline. Thus, excessive neuronal excitability could be a potential target for therapeutic intervention.

### Conclusions and future directions

Emerging evidence shows that abnormally elevated neuronal activity is a common functional feature of AD that is associated with greater cognitive decline. There are probably a few reasons for this: increased neuronal activity stimulates the spread of amyloid and tau pathology [72, 73, 84, 85], and in the long term it can lead to excitotoxicity and cell death [48]. A more sophisticated explanation may be that neuronal networks require fine regulation to function appropriately, as changes in the firing properties of a single cortical neuron are sufficient to elicit changes in behavior [156, 157]. Neuronal hyperactivity can also render neurons unable to encode spatial information or discriminate visual stimuli [158, 159]. Considering AD is a multifactorial disease, it is likely that several factors, rather than a single cause, act in conjunction to disrupt neuronal excitability regulation. In this review, we discussed some of these factors: excessive levels of cytoplasmic Ca^2+^ and glutamate; proteins commonly associated with AD, such as Aβ, apoE4, and tau; and the dysregulation of inhibitory interneuron and glial cell function.

Many questions remain to be answered about hyperexcitability in AD. Clinical fMRI studies demonstrate specific brain areas are hyperactive in patients with MCI, as well as asymptomatic offspring of AD patients and at-risk individuals, while AD patients show brain hypoactivity. However, the exact timeline for these events is unclear. When does hyperactivity start, and at which point does it begin to decrease? Would medical intervention be beneficial and if so at what point of disease progression or when would it be too late? Other studies highlight neuronal hyperexcitability as a predictor of subsequent hypoexcitability, but the mechanisms causing this shift in neuronal activity are not fully characterized. How does hyperexcitability eventually lead to reduced neuronal activity? To answer these questions we need to consider which models should be used to investigate this phenotype. Ideally, we want a model that is able to recapitulate what we see in human studies: a shift from hyper- to hypoexcitability in specific brain areas. Since all models have advantages and disadvantages, there is strength in combining information from human studies with animal and cell models of AD.

Human studies have been crucial to confirm that hyperactivity is not an artifact of laboratory models but also occurs in patients. Yet, clinical studies are observational and limited in the mechanistic information that can be tested in living patients. A vast amount of information has come from animal studies, which permit the manipulation of variables and the investigation of effects on an entire organism. This model, however, is disadvantaged by species differences. Recent data has been provided via human iPSCs, which give us the opportunity to study cells from AD patients, and to understand how specific mutations or allele variants affect cellular function. The downside is that neuronal cultures do not recapitulate the entire brain and its complex networks. Compared to organoids, 2D culture systems are an easier and faster method to study the mechanisms governing hyperexcitability. They are also well-established, meaning there is more data available for comparison. However, they lack the cytoarchitecture of the brain, and studies have shown that cells isolated from the brain exhibit significant changes in gene expression when in two-dimensional cell cultures [160, 161]. This disadvantage can be overcome with 3D cultures/organoids [162], which allow more complex interactions between cells and the interaction between cells and the extracellular matrix. They also provide a better spatial organization of the cells [105, 163]. Nonetheless, this model still presents drawbacks. The cultures are labor-intensive, more costly, and can show high variability between batches. It can also take months for the neurons within the organoids to mature [164]. Overcoming these difficulties is therefore critical for future studies to continue providing reliable data on neuronal hyperexcitability.

The use of fMRI to detect elevated brain activity may help to identify individuals at risk before symptom onset. However, because hyperexcitability is not exclusive to AD, but observed in other neurodegenerative diseases, such as ALS [154], its use as a biomarker for AD should be done in combination with other diagnostic methods currently available. As we discussed in this review, other cell types, such as inhibitory neurons and glial cells, influence neuronal excitability. Coculture systems of iPSC-derived neurons and glia combining healthy and diseased cells can give us more information on the role of each cell type [165]. In both cells and animals, gene-editing techniques, such as CRISPR-Cas9, can be used to study the role of specific cell subtypes or specific proteins and receptors. Another approach to investigate which channels are dysfunctional in the AD brain is microtransplanting native receptors from human postmortem tissue into frog oocytes [166–168]. This technique enables the study of the electrophysiological properties of disease-related receptors and can be used to validate the efficiency of novel drugs in human receptors. A deeper understanding of the specific cell subtypes and channels altered in AD will permit the development of drugs with minimal off-target effects.

Ultimately, understanding the circuits that are affected in AD, as well as their composite cell types, will be important for pinpointing the changes in neuronal excitability and how they drive or interact with pathology. To achieve this, the continuation of clinical and preclinical studies is essential to gather the information that can be translated into novel diagnostic and/or treatment strategies for AD.
